# High Efficacy on the Death of Breast Cancer Cells Using SPMHT with Magnetite Cyclodextrins Nanobioconjugates

**DOI:** 10.3390/pharmaceutics15041145

**Published:** 2023-04-04

**Authors:** Costica Caizer, Isabela Simona Caizer-Gaitan, Claudia Geanina Watz, Cristina Adriana Dehelean, Tiberiu Bratu, Codruța Soica

**Affiliations:** 1 Department of Physics, Faculty of Physics, West University of Timisoara, 300223 Timisoara, Romania; 2 Department of Plastic and Reconstructive Surgery, Faculty of Medicine, “Victor Babes” University of Medicine and Pharmacy of Timisoara, 300041 Timisoara, Romania; 3 Department of Clinical Practical Skills, Faculty of Medicine, “Victor Babes” University of Medicine and Pharmacy of Timisoara, 300041 Timisoara, Romania; 4 Department of Pharmaceutical Physics, Faculty of Pharmacy, “Victor Babes” University of Medicine and Pharmacy of Timisoara, 300041 Timisoara, Romania; 5 Research Centre for Pharmaco-Toxicological Evaluation, “Victor Babes” University of Medicine and Pharmacy of Timisoara, 300041 Timisoara, Romania; 6 Department of Toxicology and Drug Industry, Faculty of Pharmacy, “Victor Babes” University of Medicine and Pharmacy Timisoara, 300041 Timisoara, Romania; 7 Department of Pharmaceutical Chemistry, Faculty of Pharmacy, “Victor Babes” University of Medicine and Pharmacy of Timisoara, 300041 Timisoara, Romania

**Keywords:** in vitro SPMHT, ferrimagnetic nanobioconjugates, MCF-7 human breast adenocarcinoma cells, Alamar Blue analysis, cell viability

## Abstract

In this study, we present the experimental results obtained in vitro on the human breast adenocarcinoma cell line (MCF-7) by applying superparamagnetic hyperthermia (SPMHT) using novel Fe_3_O_4_-PAA–(HP-γ-CDs) (PAA is polyacrylic acid and HP-γ-CDs is hydroxypropyl gamma-cyclodextrins) nanobioconjugates previously obtained by us. In the in vitro SPMHT experiments, we used concentrations of 1, 5 and 10 mg/mL of Fe_3_O_4_ ferrimagnetic nanoparticles from Fe_3_O_4_-PAA–(HP-γ-CDs) nanobioconjugates suspended in culture media containing 1 × 10^5^ MCF-7 human breast adenocarcinoma cells. The harmonic alternating magnetic field used in the in vitro experiments that did not affect cell viability was found to be optimal in the range of 160–378 Gs and at a frequency of 312.2 kHz. The appropriate duration of the therapy was 30 min. After applying SPMHT with these nanobioconjugates under the above conditions, MCF-7 cancer cells died out in a very high percentage, of until 95.11%. Moreover, we studied the field up to which magnetic hyperthermia can be safely applied without cellular toxicity, and found a new upper biological limit H × f ~9.5 × 10^9^ A/m⋅Hz (H is the amplitude and f is the frequency of the alternating magnetic field) to safely apply the magnetic field in vitro in the case of MCF-7 cells; the value was twice as high compared to the currently known value. This is a major advantage for magnetic hyperthermia in vitro and in vivo, because it allows one to achieve a therapy temperature of 43 °C safely in a much shorter time without affecting healthy cells. At the same time, using the new biological limit for a magnetic field, the concentration of magnetic nanoparticles in magnetic hyperthermia can be greatly reduced, obtaining the same hyperthermic effect, while at the same time, reducing cellular toxicity. This new limit of the magnetic field was tested by us in vitro with very good results, without the cell viability decreasing below ~90%.

## 1. Introduction

Magnetic hyperthermia (MHT) of cancer is one of the most promising alternative methods in cancer therapy [1,2,3,4,5,6,7,8,9], aiming at the total destruction of the malignant tumor with minimal or even no toxicity on the healthy tissue. This non-invasive technique uses biocompatible magnetic nanoparticles and an alternating magnetic field with a small amplitude (kA/m—several tens of kA/m) and a frequency in the range of hundreds of kHz, which leads to the heating of the nanoparticles in the tumor to a temperature of ~43–45 °C [1,8,10]. Thus, at this temperature, tumor cells are thermally destroyed by apoptosis and/or necrosis. Therefore, the therapeutic effect is natural, without radiation and chemical drugs that have a high degree of toxicity on the living organism.

Kandasamy et al. [11], by applying magnetic hyperthermia in vitro, reported the death of MCF-7 tumor cells at a percentage of approximately 90%, using superparamagnetic Fe_3_O_4_ nanoparticles of 9 nm diameter covered with dual surfactants of TA–ATA (terephthalic acid–aminoterephthalic acid). The concentration used in the experiment was 1 mg/mL of magnetic nanoparticles in suspension, and the frequency of the magnetic field was 751.5 kHz in the admissible biological range.

A similar result on MCF-7 tumor cells was reported by Bhadwaj et al. [12], using magnetic hyperthermia with nanoparticles of Mn_0.9_Zn_0.1_Fe_2_O_4_ having a size of 11.3 nm and covered with lauric acid.

Magnetic hyperthermia in vivo also leads to very promising results for cancer therapy. Thus, Alphadery et al. [13] applied magnetic hyperthermia on breast tumors xenografted under the skin of mice using magnetosome chains in suspension until a 10 mg/mL concentration of iron oxide nanoparticles was reached. After applying the therapy several times for 20 min. with a field of 40 mT and frequency of 198 kHz, using doses of 0.1 mL injected each time in the center of the ~100 mm^3^ tumor, the tumors in several mice were completely reduced.

Furthermore, Wang et al. [14] used in vivo magnetic hyperthermia with HPMC/Fe_3_O_4_ nanoparticles (HPMC is hydroxyl-propyl methyl cellulose) with 10–50 nm Fe_3_O_4_ nanoparticles, by intratumoral injection, for the therapy of tumor-bearing mice. After 14 days of treatment by exposure to the magnetic field with a frequency of 626 kHz, a high-efficiency ablation of tumors was obtained.

Currently, MagForce AG [15] uses magnetic hyperthermia with Fe_3_O_4_ nanoparticles of 12 nm covered with aminosilanes, for the treatment of glioblastoma and prostate cancers in preclinical trials, with good results.

Although in principle, the magnetic hyperthermia technique seems easy to approach, it raises very complex aspects that must be well-clarified and overcome before being safely applied in clinical trials, such as the following: (i) finding the most suitable magnetic nanoparticles (NPs) for this type of therapy; (ii) their high biocompatibility with the biological tissue (lack of cellular toxicity), but also (iii) finding the parameters of the alternating magnetic field; (iv) the most suitable conditions for the practical implementation of magnetic hyperthermia; and (v) the most suitable therapeutic plan. All of these aspects are necessary to obtain the maximum effectiveness on the death of tumor cells, and to minimize the toxicity on normal cells. Until now, there are many studies that have been conducted in this direction to maximize one or more of the aspects listed above (i)–(v) [1,2,9], but they did not establish which are the most suitable to be used in magnetic hyperthermia for in vivo and clinical trials [1,16].

Considering the above aspects, we previously proposed the use of Fe_3_O_4_ nanoparticles coated with gamma cyclodextrins (γ-CDs) [17,18] to increase the effectiveness and reduce cellular toxicity to a minimum. We also found the optimal conditions [19] in which superparamagnetic hyperthermia (SPMHT) can be applied for maximum effectiveness on tumors. These aspects were studied by us both theoretically [17,19] and experimentally [18], and the recent experimental results showed the possible successful application of nanobioconjugates of Fe_3_O_4_-PAA–(HP-γ-CDs) (PAA is polyacrylic acid, HP-γ-CDs is hydroxypropyl gamma cyclodextrin) on the destruction of tumors through SPMHT [18], which is more effective than MHT [20]. We used γ-CDs for the bioconjugation of Fe_3_O_4_ magnetic nanoparticles, due to their advantages for magnetic hyperthermia. Cyclodexdrins are very suitable for coating nanoparticles, in terms of stability over time and their biocompatibility with magnetic hyperthermia and drug delivery [21]; they are natural cyclic oligosaccharides without toxicity that are used in pharmaceutics, cosmetic products and biomedicine [22]. Moreover, due to their very reduced thickness on the surface of magnetic nanoparticles, an increase in efficiency in magnetic hyperthermia was obtained by increasing the concentration of nanoparticles in an injectable dispersion targeting tumors. Moreover, cyclodextrins make inclusion complexes possible by encapsulating anticancer drugs in their poorly hydrophilic central cavities (as encapsulated doxorubicin), which in the future would also allow the realization of a double therapy: magnetic hyperthermia followed by the release of the local drug in the tumor, thereby increasing the effectiveness of therapy on tumors.

Using the optimal conditions previously established by us [17,18,19] for the efficient implementation of SPMHT, in this study we present the experimental results that we obtained in vitro by applying SPMHT using Fe_3_O_4_-PAA–(HP-γ-CDs) nanobioconjugates on MCF-7 human breast adenocarcinoma cells. We chose this cancer for in vitro testing due to its high incidence in women and high mortality rate, according to the World Health Organization [23]. We mainly present three innovative experimental results that are very important for the advancement of the field of application of superparamagnetic hyperthermia in MCF-7 breast cancer therapy. The following results pertain to the increase in its efficacy on the destruction of tumors and the reduction in toxicity:

(i) The use in superparamagnetic hyperthermia experiments in vitro of biocompatible Fe_3_O_4_-PAA–(HP-γ-CDs) magnetic nanobioconjugates previously obtained by us, which have not been used so far in the superparamagnetic hyperthermia of tumors;
(ii) Experimental demonstration of the high efficacy of SPMHT using Fe_3_O_4_-PAA–(HP-γ-CDs) nanobioconjugates via testing in vitro on the MCF-7 breast cancer cell line, with very good results regarding the death of tumor cells at a very high percentage (up to approximately 95%);
(iii) Based on our experimental results on MCF-7 cells, we established a new upper biological limit for the magnetic field without cellular damage for which magnetic hyperthermia can be safely applied on MCF-7 cells: we found that the maximum admissible biological limit can be extended to a double value compared to the previously known one, with superior advantages in the practical implementation of SPMHT for tumors to increase its efficacy and reduce cellular toxicity.

## 2. Materials and Methods

### 2.1. Magnetic Nanobioconjugates Used for Testing SPMHT on MCF-7 Breast Cancer Cells

#### 2.1.1. Synthesis Method of Nanobioconjugates

For in vitro SPMHT on MCF-7 breast tumor cells, we used our previously obtained Fe_3_O_4_-PAA–(HP-γ-CDs) nanobioconjugates [18]. First, ferrimagnetic Fe_3_O_4_ nanoparticles were obtained by the chemical co-precipitation method; then, the polyacrylic acid (PAA) adsorbed on their surface [24,25] was used for binding (bioconjugation) with hydroxypropyl-gamma-cyclodextrins (HP-γ-CDs), in the second stage, following the procedure described in detail in reference [18]. We used the branched cyclodextrins HP-γ-CDs because by the molecular docking method, we previously showed that this nanobiostructure is the most stable. At the same time, cyclodextrins ensure a very good biocompatibility of nanoparticles with the biological environment [26].

#### 2.1.2. Characterization Techniques of Nanobioconjugates

For the characterization of nanobioconjugates, we used X-ray diffraction (XRD), Fourier transform infrared spectroscopy (FT-IR), high-resolution transmission electron microscopy (HR-TEM) and dynamic light scattering (DLS) techniques, presented in detail in our previous reference [18].

XRD was used to determine the crystalline phases and the average size of the nanocrystallites. A Rigaku UltimaIV Diffractometer with Cu Kα radiation was used for this.

FT-IR was used for the study of ferrite formation and the determination of specific Me–O bonds (Me: metal, O: oxygen) in the magnetite structure. A Shimadzu IR Affinity-1S spectrophotometer was used in the 400–4000 cm^−1^ range.

The morphology of the sample, and the size and distribution of the nanoparticles was studied via HR-TEM, using a Hitachi TEM system (HT7700) with 0.2 nm resolution.

Using the Vasco Particle Size Analyzer, the average hydrodynamic diameter of the nanobioconjugates in dispersion and their distribution was determined using DLS.

### 2.2. Magnetization of the Fe_3_O_4_-PAA–(HP-γ-CDs) Nanobioconjugates

The magnetization of the Fe_3_O_4_-PAA–(HP-γ-CDs) nanobioconjugates and their magnetic behavior in the external magnetic field was carried out with the equipment described in reference [27]. The sample was in the form of nanobioconjugate powder, with a mass of 299.4 mg and a packing volume fraction of 0.62; the applied field was ~1900 Oe (~151 kA/m in SI units). The applied magnetic field was much higher than the one used in the magnetic hyperthermia experiments. The magnetization was determined as a specific value, in emu/g unit. The magnetic behavior in the field of nanobioconjugates was determined from the magnetization curve recorded towards magnetic saturation.

### 2.3. In Vitro Magnetic Hyperthermia Experiment

#### 2.3.1. MCF-7 Cancer Cell Line

The MCF-7 cell line was acquired from the American Type Culture Collection (ATCC, Manassas, VA, USA) as a frozen vial. The cell line was grown in Eagle’s Minimum Essential Medium (EMEM) enriched with 15% FCS (Fetal Calf Serum), and supplemented with an antibiotic mixture of penicillin/streptomycin (100 U/mL penicillin and 100 μg/mL streptomycin) to avoid possible microbial contamination. The cells were passaged every two to three days using trypsin/EDTA until the confluence of the cells reached approximately 80–85%. The cell cultures were maintained under standard conditions: a humidified atmosphere enriched with 5% CO_2_ and a temperature of 37 °C, using a Steri-Cycle i160 incubator (Thermo Fisher Scientific, Inc., Waltham, MA, USA).

#### 2.3.2. In Vitro Magnetic Hyperthermia Protocol

To evaluate the cytotoxic effect induced by the Fe_3_O_4-_PAA–(HP-γ-CDs) suspension under magnetically induced hyperthermia, a slightly modified protocol of Quinto et al. was employed [28]. Briefly, when the MCF-7 cells reached a confluence higher than 80%, the cells were split using trypsin/EDTA solution. Afterwards, the cells were counted using the trypan blue exclusion technique. Based on this step, a cell suspension of 1 × 10^5^ cells/mL was obtained by dilution of the cell pellet in cell medium. The sample-free cell suspension and cell suspensions containing Fe_3_O_4-_PAA–(HP-γ-CDs) of different concentrations (1, 5, 10 mg/mL) were inserted in 2 mL vials, and were further exposed to alternating magnetic fields (AMF) (312.2 kHz and different fields 160, 200 and 378 Gs).

#### 2.3.3. Alamar Blue Testing

To quantify the effect induced by the AMF on sample-free cell suspensions, and to evaluate the cytotoxic potential of the Fe_3_O_4_-PAA–(HP-γ-CDs) suspension at three different concentrations (1, 5 and 10 mg/mL) on the human adenocarcinoma MCF-7 cell suspension, the Alamar Blue colorimetric test was employed. In brief, after AMF exposure of the cell suspension ended, 2 × 10^4^ cells/well were seeded in 96-well plates and were furter maintained in a humidified atmosphere at 37 °C and 5% CO_2_ until an interval of 24 h was reached. Afterwards, the cells were rinsed three times with phosphate buffer saline (PBS) to avoid possible interference with the Alamar Blue reagent, as previously described [18]. This washing step was followed by the addition of 200 µL of culture medium in each well. The control cells were exposed only to culture media under standard conditions (37 °C). Afterwards, Alamar Blue reagent was added into each well to a final concentration of 0.01%. The cell viability percentage was quantified at 3 h post-addition of the Alamar Blue reagent by measuring the absorbance of the wells at two different wavelengths (570 nm and 600 nm) with a microplate reader (xMark^TM^ Microplate, Bio-Rad Laboratories, Hercules, CA, USA), as previously described [29].

#### 2.3.4. Magnetic Hyperthermia Experiment

The magnetic hyperthermia experiments were carried out using specialized equipment (F3 Driver, nB, Zaragoza, Spain) for this type of experiment, with 3 kW of power and a varying frequency (f) and magnetic field amplitude (H). In our experiments, we used a frequency of 312.2 kHz, and fields of 160 (12.73), 200 (15.92) and 378 Gs (30.08 kA/m in S.I. units), depending on the concentrations of Fe_3_O_4_ nanoparticles from Fe_3_O_4_-PAA–(HP-γ-CDs) nanobioconjugates suspended in the cell culture medium, which allowed us to obtain a magnetic hyperthermia therapy temperature of 42.9 °C, without affecting the cells (see Section 3.3.1). The samples used for the magnetic hyperthermia experiments were in fact MCF-7 cell suspensions (density of 1 × 10^5^ cells/mL) that contained Fe_3_O_4_-PAA–(HP-γ-CDs) nanobioconjugates at concentrations of 1, 5 and 10 mg/mL equivalents in F_3_O_4_ NPs. These samples were pipetted into different vials of 2 mL capacity, and were furthermore individually exposed to a magnetically induced hyperthermia for a period of 30 min using adiabatic conditions, and employing the specialized inductor coil of the equipment. The temperature of the samples obtained under magnetic hyperthermia was measured using a precision fiber optic sensor.

#### 2.3.5. Data Representation and Statistical Analysis

GraphPad Prism 9 version 9.3 (GraphPad Software, San Diego, CA, USA) was used for data representation and statistical analysis. The results are presented as mean values ± standard deviations (SDs). One-way ANOVA was performed to obtain the statistical differences, followed by Tukey’s multiple comparison test.

## 3. Results and Discussion

### 3.1. The Fe_3_O_4_-PAA–(HP-γ-CDs) Nanobioconjugates Data

The Fe_3_O_4_-PAA–(HP-γ-CDs) nanobioconjugates obtained by us [18] were formed by ferrimagnetic nanoparticles of Fe_3_O_4_ (magnetite), as determined with X-ray diffraction (XRD) and high-resolution transmission electron microscopy (HR-TEM). They were approximately spherical, with a size (average diameter) of ~16 nm, and covered by bioconjugation with hydroxypropyl gamma-cyclodextrins (HP-γ-CDs) by means of the polyacrylic acid (PAA) biopolymer [24,25]; these were evidenced by Fourier transform infrared spectroscopy (FT-IR), which formed nontoxic nanobioconjugates of ~20 nm in mean diameter, as determined by dynamic light scattering (DLS) [18]. These nanobioconjugates were very suitable for increasing the efficacy of SPMHT in the destruction of tumor cells, due to the optimal size of the magnetic nanoparticles of magnetite, and the existence of the thin organic layer with which they were covered. This allowed for increasing the concentration of nanobioconjugates of Fe_3_O_4_-PAA–(HP-γ-CDs) in the suspension to be injected into the tumor, thus increasing the hyperthermic effect. At the same time, using the organic layer with cyclodextrins of the HP-γ-CDs type that formed polyacrylate functionalized complexes of Fe_3_O_4_-PAA–(HP-γ-CDs), the nanobioconjugates obtained by us and used in the in vitro experiments for magnetic hyperthermia of tumors were nontoxic for healthy cells, as cyclodextrins are natural oligosaccharides [26].

### 3.2. Magnetic Behavior of Fe_3_O_4_-PAA–(HP-γ-CDs) Nanobioconjugates

**Figure 1:**
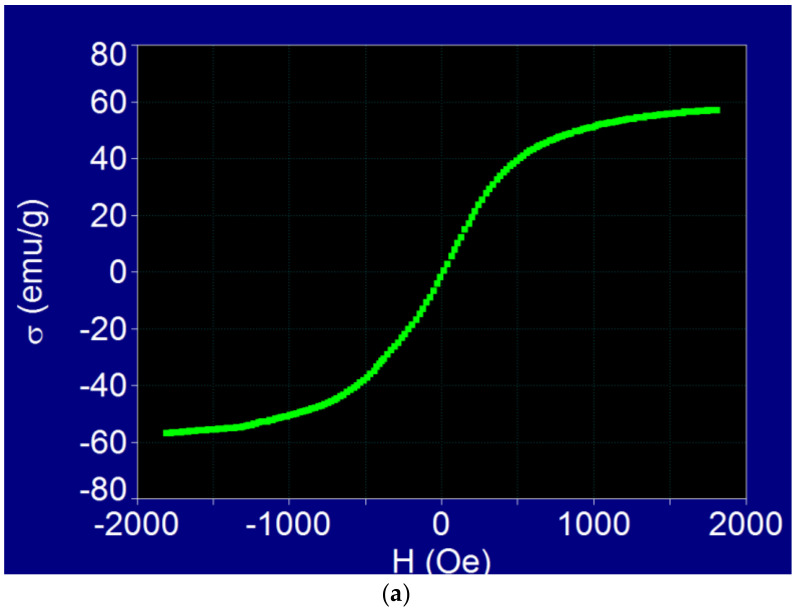

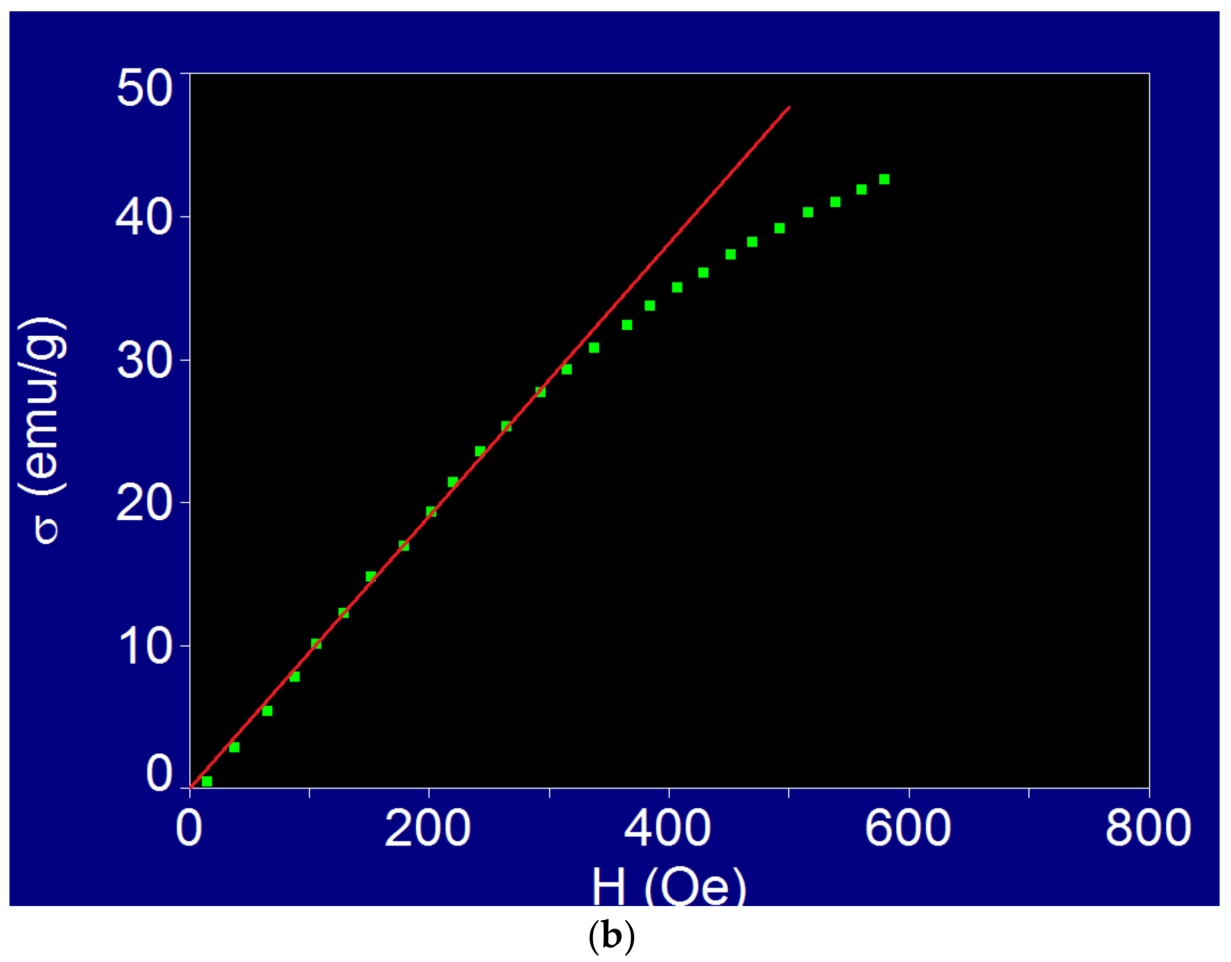
Specific magnetization (σ) loop to (**a**) magnetic saturation and (**b**) low magnetic field (H) of Fe_3_O_4_ nanoparticles from Fe_3_O_4_-PAA–(HP-γ-CDs) nanobioconjugates; the green points are experimental data, and the red line is the linear fit function (r^2^ = 0.997).

The experimental specific magnetization curves towards saturation and a low magnetic field in the case of Fe_3_O_4_ nanoparticles from the Fe_3_O_4_-PAA–(HP-γ-CDs) nanobioconjugates powder sample are shown in Figure 1.

The shape of the magnetization curve from Figure 1a shows a very important aspect, namely, that the magnetic behavior in the external magnetic field of the Fe_3_O_4_-PAA–(HP-γ-CDs) nanobioconjugates, having a ferrimagnetic Fe_3_O_4_ nanoparticle size (average diameter) of ~16 nm, and an average hydrodynamic diameter of ~20 nm of the superparamagnetic type: the magnetization curve was without hysteresis, and the coercive field was zero. The lack of hysteresis in such a magnetic field, which was much higher than the one used in the magnetic hyperthermia experiments, confirms the fact that the hyperthermic effect was obtained in this case as a result of superparamagnetic hyperthermia (SPMHT), as we presented theoretically in references [17,19]. This result was very important for the practical implementation of magnetic hyperthermia because the loss power, including the heating temperature obtained in superparamagnetic hyperthermia (SPMHT), was higher than that in magnetic hyperthermia (MHT) [30].

Moreover, the specific saturation magnetization (σ) of Fe_3_O_4_ nanoparticles from Fe_3_O_4_-PAA–(HP-γ-CDs) nanobioconjugates was in this case quite high, being 56.95 emu/g, compared to that of bulk Fe_3_O_4_, which is 92 emu/g [31], despite the sample being in a powder state (the magnetization of powders is significantly lower). At the same time, the Fe_3_O_4_ nanoparticles, whose saturation magnetization was lower due to surface effects [32,33], were additionally covered with the PAA–(HP-γ-CDs) organic layer, which further reduced the saturation magnetization. The high value of the specific saturation magnetization in this case was due to the reduced thickness of the organic layer of PAA–(HP-γ-CDs) on the surface of the nanoparticles, which was only ~3.2 nm; this determined a volume packaging fraction [34] that was significantly higher than in the case of other larger biostructures (e.g., liposomes, which have much larger sizes of tens or hundreds of nm) [26].

The experimental specific initial magnetic susceptibility (mass susceptibility) evaluated from the magnetization curve registered at low magnetic fields (Figure 1b) had a value of 95.4 × 10^−3^ emu/g Oe (or 6.28 in SI units for initial magnetic susceptibility (volume susceptibility), considering the density of Fe_3_O_4_ nanoparticles). This value was determined by fitting a linear function (red line in Figure 1b) with the experimental data (green points) obtained for low fields, and then determining the slope of fit line. The value found for the initial magnetic susceptibility is very good for magnetic hyperthermia.

Thus, the high value of the magnetization and magnetic susceptibility of magnetic nanoparticles from the nanobioconjugates allowed us to obtain a high hyperthermic effect in a short time, with the specific loss power [17] leading to the heating of the nanoparticles in proportion to this observables [19].

These results, in the case of our nanobioconjugates indicated (a) superparamagnetic behavior of Fe_3_O_4_-PAA–(HP-γ-CDs) nanobioconjugates with Fe_3_O_4_ nanoparticles of ~16 nm, and (b) a high saturation magnetization and initial magnetic susceptibility of nanoparticles that was very important for the in vitro experiments (which are presented in Section 3.3.2) to efficiently obtain the hyperthermic effect (reaching a temperature of ~43 °C) in the shortest possible time, so that healthy cells are not affected.

### 3.3. In Vitro Testing on Breast Cancer Cells of SPMHT with Fe_3_O_4_-PAA–(HP-CDs) Nanobioconjugates

For the in vitro testing of SPMHT on MCF-7 breast cancer cells, we used three suspensions of Fe_3_O_4_-PAA–(HP-γ-CDs) nanobioconjugates suspended in the culture medium, with concentrations of Fe_3_O_4_ magnetic nanoparticles of 1, 5 and 10 mg/mL. The establishment of these concentrations was based on our previous results [18], which showed that at these concentrations, the viability of healthy cells was not affected. The values used for the magnetic field parameters (amplitude H and frequency f) are those shown in Section 3.3.1 and Section 3.3.2 However, in our in vitro SPMHT experiments, we used automatic (electronic) tuning of the amplitude of the magnetic field without exceeding the admissible biological limit, in order to obtain a temperature of 42.9 °C required for hyperthermia; hence, we maintained this temperature throughout the duration of the experiments (see Section 3.3.2). The duration of each in vitro SPMHT experiment was 30 minment.

#### 3.3.1. The Effect of Magnetic Field on Cell Viability of MCF-7 Breast Cancer Cells

An initial experiment we performed was to see if the amplitude of the magnetic field that was going to be used in our in vitro SPMHT experiments could affect MCF-7 breast cancer cells. For this experiment, the frequency of the magnetic field was established at 312.2 kHz, and a cell suspension of MCF-7 cells to a density of 1 × 10^5^ cells/mL was used under standard conditions (37 °C). We also used the case of cellular distribution in suspension, putting the cell culture in the culture medium in 2 mL vial bottles, which is more realistic than the cultures in plates (2D) because this is closer to the in vivo experiments for the tumor animal model [35]. Magnetic fields of 160, 200 and 378 Gs were applied to the samples with MCF-7 cancer cells (three identical samples). These values of the magnetic field were used later in the SPMHT experiments with Fe_3_O_4_-PAA–(HP-γ-CDs) nanobioconjugates on MCF-7 cancer cells (see Section 3.3.2). We set these values for the magnetic field because they reached the 42.9 °C temperature required for magnetic hyperthermia for the 1, 5 and 10 mg/mL nanobioconjugate preparations (mass of Fe_3_O_4_ ferrite nanoparticles from Fe_3_O_4_-PAA–(HP-γ-CDs)) nanobioconjugates dispersed in 1 mL of PBS).

**Figure 2:**
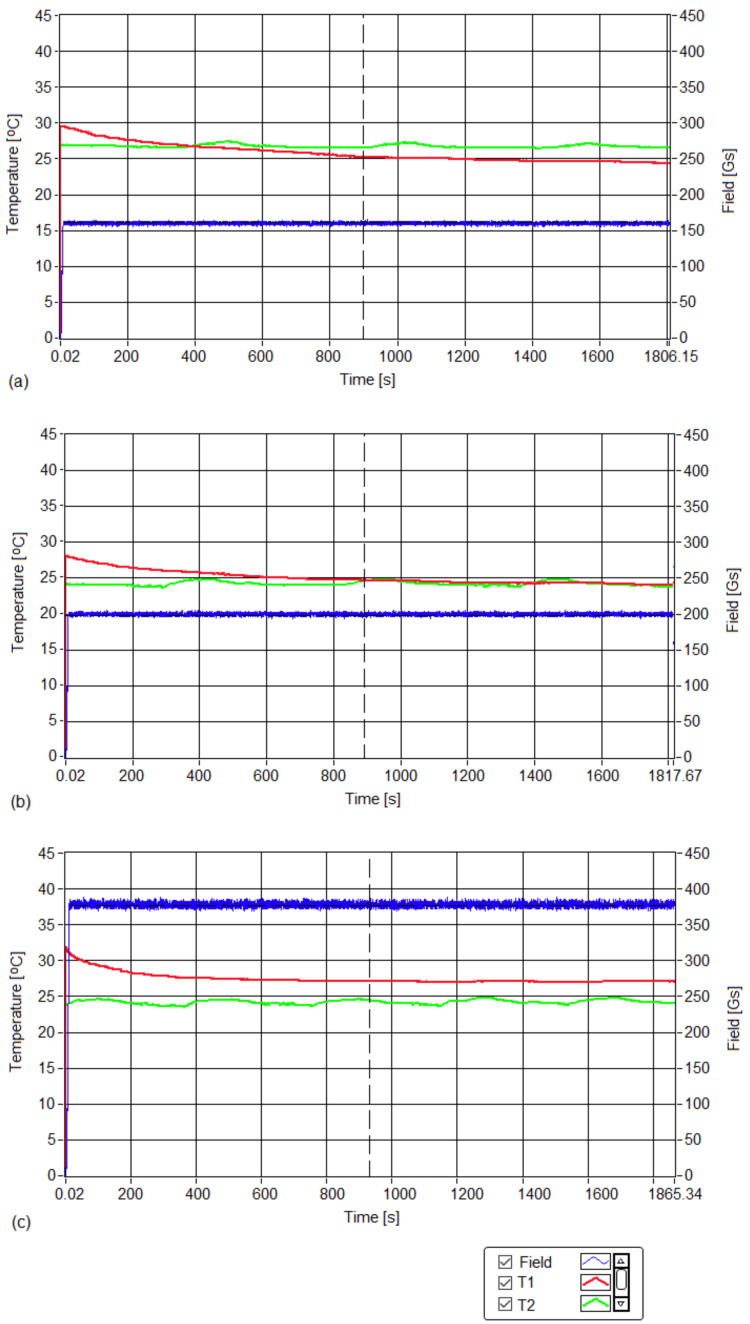
Time diagrams for the temperature of the cell culture (red curve) recorded for 30 min at magnetic field (blue curve) amplitudes of (**a**) 160, (**b**) 200 and (**c**) 378 Gs. The green curve shows the room’s temperature.

The experimental curves that show the amplitude of the applied magnetic field and the temperatures of the cell culture recorded for the three samples are in Figure 2.

**Figure 3:**
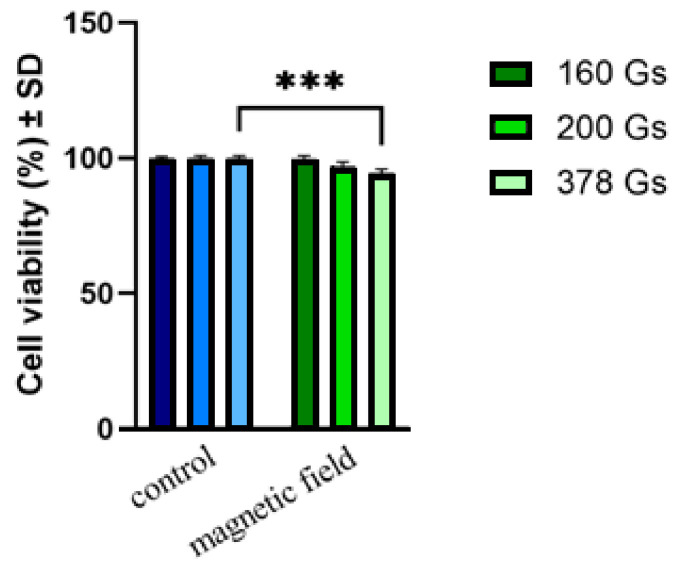
Cell viability of MCF-7 cell line post-exposure to magnetic field for a period of 30 min, at a frequency of 312.2 kHz and different amplitudes (160, 200 and 378 Gs). The viability percentages were normalized to control cells (cells treated only with culture medium and maintained under standard conditions (37 °C)). Data are represented as mean values ± standard deviations (SDs). One-way ANOVA analysis was applied to determine the statistical differences followed by Tukey’s post-test (*** *p* < 0.001).

The obtained experimental results showed the following:

(i) No hyperthermic effect (>40 °C) was obtained; the temperatures of cell cultures T1 (red curve) in all three cases remained close to room temperature T2 (green curve) for all three magnetic fields throughout the 30 min. experiment. Thus, in the absence of magnetic nanoparticles in samples with MCF-7 cancer cells, the alternating magnetic field with a frequency of 312.2 kHz and amplitudes in the range of 160–378 Gs did not lead to an increase in the temperature of the cells;
(ii) For all three values of the magnetic field (160, 200 and 378 Gs), the cell viabilities (Figure 3) of sample-free MCF-7 cancer cells exposed to magnetic fields for 30 min. were practically unaffected, the value of viabilities (Table 1) remaining in the acceptable range of ISO 10993-5 (the International Organization for Standardization) (ISO 10993-5:2009, reviewed and confirmed in 2017) (with a possible viability decrease of 30%) [36].

The results obtained following the Implementation of the Alamar Blue test revealed that the viability of the MCF-7 breast cancer line was only slightly affected by exposure to the magnetic field for values greater than 200 Gs. Even after applying the highest field of 378 Gs, the viability of MCF-7 cells remained high, showing a rate of 94.50 ± 1.42%. Increasing the magnetic field from 160 to 378 Gs only led to a very small decrease in cell viability, namely, ~3% for the 200 Gs field, and ~5% for the 378 Gs field. For the smallest field, 160 Gs, the cell viability remained at practically 100%, within the limits of experimental error.

**Figure 4:**
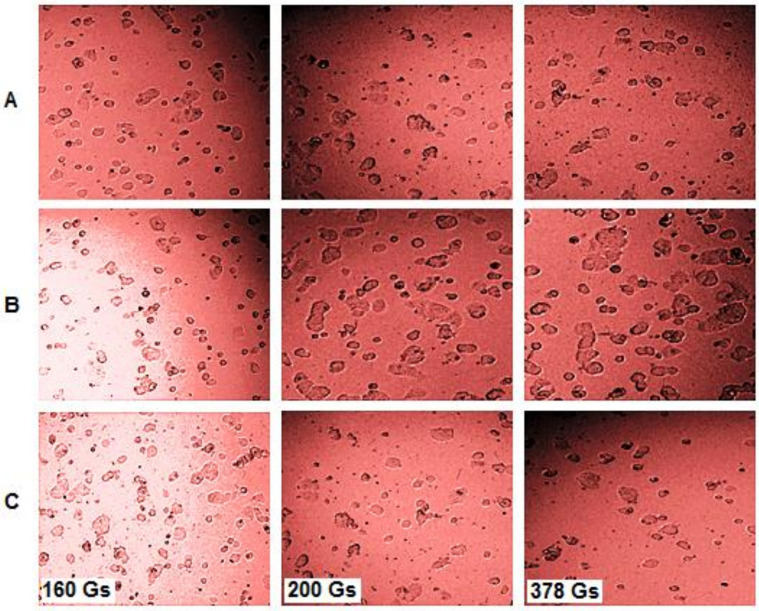
Morphological aspects (nucleus and cytoskeleton) of the MCF-7 human breast adenocarcinoma cell line in the cases of (**A**) standard conditions (37 °C), (**B**) laboratory conditions (24 °C), and (**C**) exposure to magnetic fields (30 min, frequency 312.2 kHz) at different amplitudes (160, 200 and 378 Gs).

According to ISO 10993-5 [36], related to the biological evaluation of medical devices, a sample is considered cytotoxic if the viability of the cells exposed to the sample is decreased by 30%. Hence, the results obtained showed a viability above 90%, revealing that the magnetic field applied to amplitudes in the range of 160–378 Gs did not induce a cytotoxic effect on MCF-7 cells, and could be safely used for in vitro magnetic hyperthermia experiments on human breast cancer cells. These results were also confirmed by the cellular morphological analyses presented in Figure 4.

Figure 4 presents the morphological aspects observed for the MCF-7 cell line subjected to different experimental conditions: (A) standard conditions (37 °C); (B) laboratory conditions (at room temperature of ~25–27 °C); (C) exposure to a magnetic field. In analyzing Figure 4, it can be easily stated by comparison with the cells maintained in standard conditions that (A), the cells exposed to the magnetic field (C) did not show significant alterations at the morphological level, as cells did at room temperature (laboratory conditions).

In magnetic hyperthermia the admissible upper biological limit of H × f = 5 × 10^9^ A/mHz [37], where H is the amplitude of the magnetic field and f is its frequency, was previously reported. However, in our case and under the conditions established by us, this limit was far exceeded for MCF-7 breast cancer cells, reaching a value of over ~9.5 × 10^9^ Am^−1^Hz, approximately double the value as in the case of the 378 field Gs. This is a very important experimental result obtained in our case because the field can be increased above the previously known limit (H × f = 5 × 10^9^ Am^−1^Hz); this leads to the increase in the effectiveness of magnetic hyperthermia in this case, and to a reduction in the therapy duration (to avoid possible cytotoxicity), with overall beneficial effects for this therapy.

Furthermore, an increase in the magnetic field to a value that is twice as high as the value given by the admissible biological limit known until now, is very important for magnetic hyperthermia because it can lead to a significant decrease in the concentration of magnetic nanoparticles used to obtain the same hyperthermic effect on cell tumors (reaching a temperature of 43 °C that is necessary in magnetic hyperthermia). At the same time, a significant decrease in the concentration of nanoparticles in magnetic hyperthermia has a very beneficial effect on reducing or even eliminating cytotoxicity at low concentrations.

In summary, we can say that the use of the magnetic field with a frequency of 312.2 kHz and an amplitude in the range of 160–378 Gs in magnetic hyperthermia experiments in vitro is not cytotoxic for MCF-7 tumor cells.

#### 3.3.2. SPMHT with Fe_3_O_4_-PAA–(HP-γ-CDs) Nanobioconjugates on MCF-7 Cancer Cells

**Figure 5:**
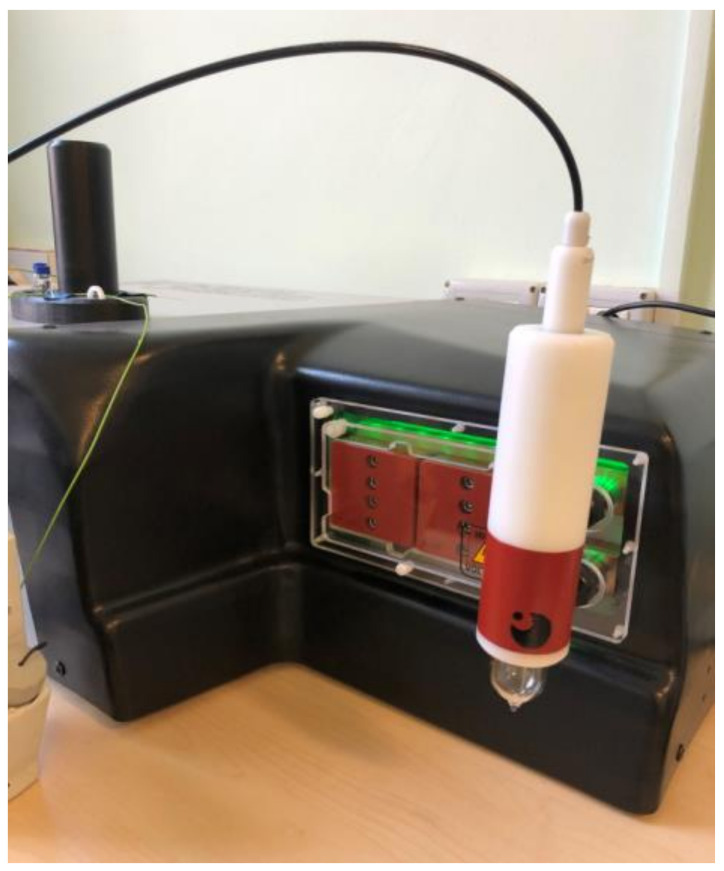
The experimental equipment for testing SPMHT in adiabatic conditions.

SPMHT on MCF-7 tumor cells using Fe_3_O_4_-PAA–(HP-γ-CDs) nanobioconjugates was tested in vitro for the concentrations of 1, 5 and 10 mg/mL (mass (in mg) of Fe_3_O_4_ ferrite nanoparticles from Fe_3_O_4_-PAA–(HP-γ-CDs) nanobioconjugates dispersed in 1 mL of culture medium), using the specialized professional equipment for magnetic hyperthermia from Figure 5, in adiabatic conditions. The cell culture in this case was very well thermally isolated from the external environment. Thus, the cell culture could be maintained at a constant temperature of 42.9 °C for the entire 30 min. of the magnetic hyperthermia experiment.

Furthermore, performing the experiment in cell suspension and not on 2D surface culture plates may have provided features that were closer to simulating real conditions encountered under in vivo experiments, such us animal models. Thus, the results of the experiments may provide more reliable data [35].

**Figure 6:**
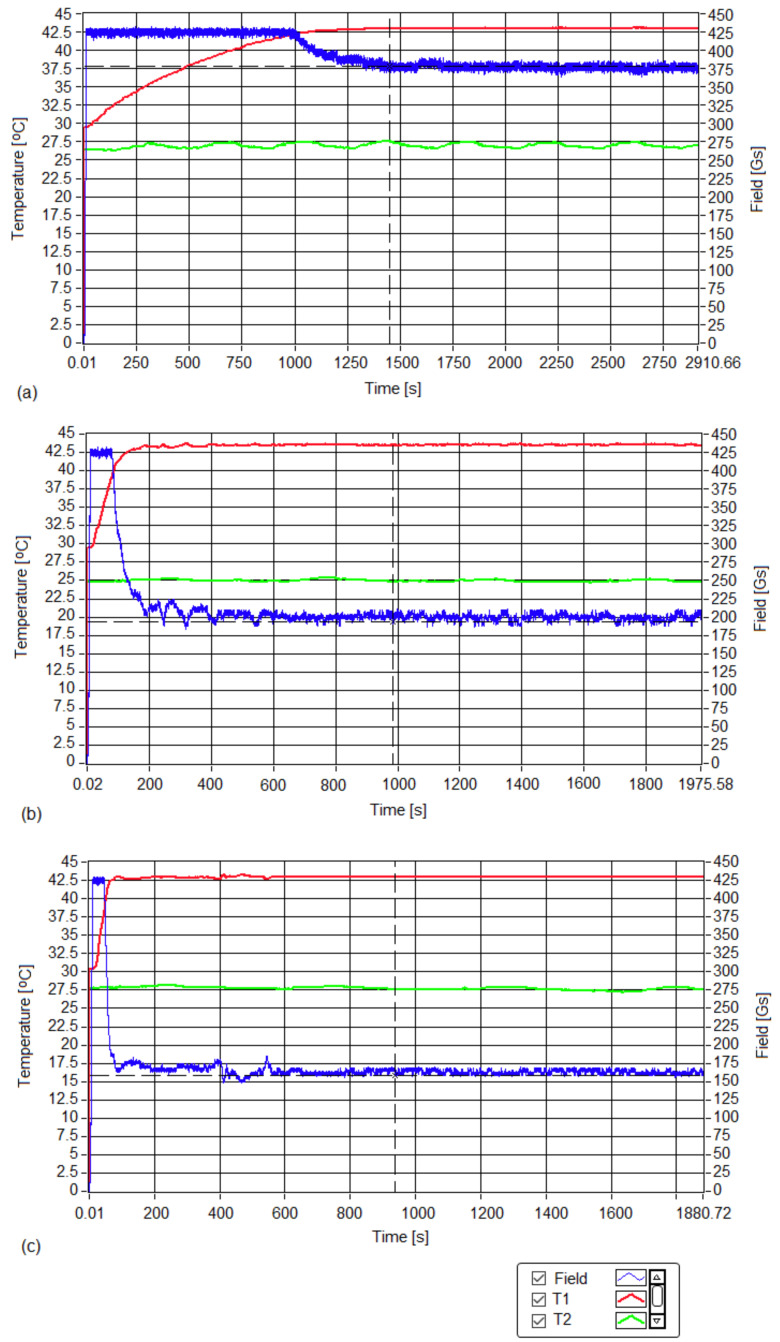
Curves for magnetic field amplitude (blue), therapy temperature T1 (red) and room temperature T2 (green) for concentrations of (**a**) 1, (**b**) 5 and (**c**) 10 mg/mL.

The experimental curves recorded during the SPMHT experiments for the three concentrations are shown in Figure 6.

The red curve shows the temperature obtained in magnetic hyperthermia, which was constant at 42.9 ± 0.1 °C throughout the duration of the 30 min. experiments for concentrations of 1 (Figure 6a), 5 (Figure 6b) and 10 mg/mL (Figure 6c). The green curve shows the room temperature during the experiments. The blue curve shows the magnetic fields during the three experiments, electronically maintained during the 30 min. of therapy so that the therapy temperature (42.9 °C) did not change (red curves in Figure 6). At the beginning, the magnetic field had a higher value that initiated a rapid increase in temperature, after which it quickly dropped to the corresponding value from the experiment, and then remained approximately constant throughout the therapy.

The experimental curves in Figure 6 demonstrate that in all three cases, the temperature required for therapy T1 (red curves) was kept constant at the treatment value of 42.9 ± 0.1 °C for the entire 30 min. therapy, through rigorous and precise (electronic) control of the amplitude of the applied magnetic field (blue curves).

At the same time, the temperature of the environment T2 (green curves) around the inductor coil where the cells were subjected to magnetic hyperthermia remained unchanged, and at a level much lower than the therapy temperature for the entire duration in the experiment; this showed that hyperthermia could effectively be applied to the cells.

The values of the magnetic field in the experiments depended on the concentrations of the magnetic nanoparticles used (1 mg/mL, 5 mg/mL and 10 mg/mL). Thus, the amplitude of the magnetic field increased when the concentration decreased. Its values are given in Table 2.

**Figure 7:**
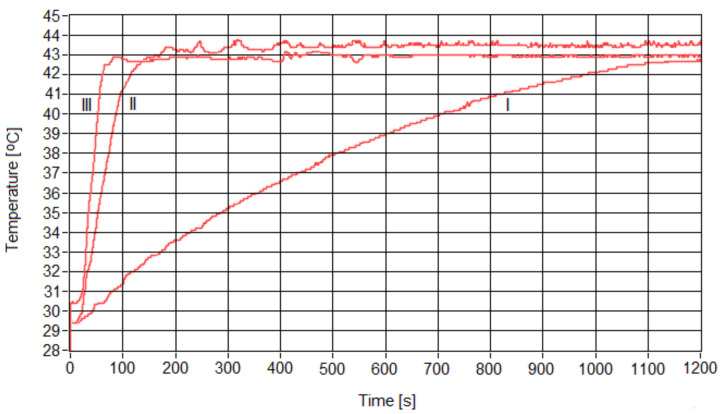
Temperature increase over time from room temperature to that corresponding to SPMHT in the cases of magnetic nanoparticle concentrations of (I) 1, (II) 5 and (III) 10 mg/mL.

However, the speed required to increase the therapy temperature (red curve) to the value corresponding to SPMHT used in our experiments varied, depending on the concentration of the magnetic nanoparticles in the nanobioconjugates (Figure 7). Thus, in the case of the more diluted sample of 1 mg/mL, the temperature reached that corresponding to magnetic hyperthermia in a longer time (20 min.) compared to the more concentrated samples, where the times were much reduced (80 s) (Table 3).

All samples with different concentrations were maintained for 30 min. (therapy duration) under identical conditions in the alternating magnetic field with a frequency of 312.2 kHz and amplitudes of 160 (12.73), 200 (15.92) and 378 Gs (30.08 kA/m) (Figure 6 and Table 2), in adiabatic conditions (Figure 5). Then, they were analyzed from the point of view of cell viability to see the hyperthermic effect obtained by SPMHT on MCF-7 breast cancer cells. This assessment was performed using the Alamar Blue calorimetric test (see next section).

### 3.4. Cell Viability Assessment of MCF-7 Cells after SPMHT Therapy via Alamar Blue Test

**Figure 8:**
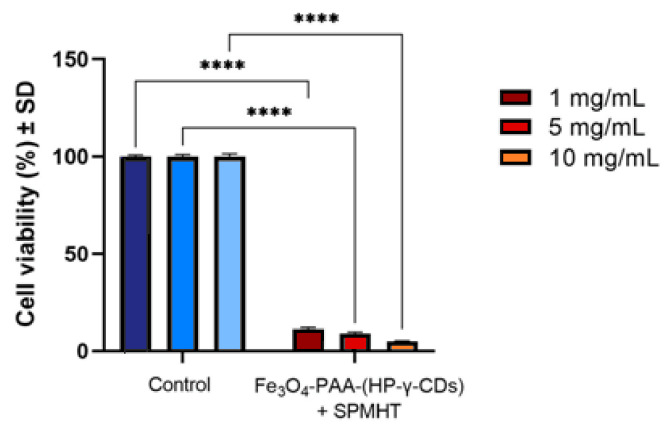
Cell viability percentages of MCF-7 human adenocarcinoma cell lines exposed to different concentrations of Fe_3_O_4_-PAA–(HP-γ-CDs) nanobioconjugates under SPMHT conditions (temperature of 42.9 °C for 30 min.) at 24 h post-stimulation. The viability percentages were normalized to control cells (cells treated only with culture medium and maintained under standard conditions (37 °C)). Data are represented as mean values ± standard deviations (SDs). One-way ANOVA analysis was applied to determine the statistical differences followed by Tukey’s post-test (**** *p* < 0.0001).

A summary of the experimental results obtained after the application of SPMHT with Fe_3_O_4_-PAA–(HP-γ-CDs) nanobioconjugates in vitro on MCF-7 breast cancer cells is shown in Figure 8. The results obtained regarding the viability of breast cancer cells after hyperthermic therapy (temperature of 42.9 °C for 30 min.) show an important decrease in MCF-7 cell populations for all three test concentrations (1, 5 and 10 mg/mL); the viability of the cells showed percentages between ~5–10%, when compared to the control value (100%) recorded under standard conditions. The highest decrease in cell viability was obtained when the higher concentration of magnetic nanoparticles (10 mg/mL) was applied, revealing that a more intense effect of MCF-7 cell death could be obtained when high concentrations of magnetic nanoparticles are used. Moreover, the IC_50_ parameter showed a good value of 0.06288 mg/mL for the magnetic nanoparticles (Figure S1).

The values of the viability percentages of MCF-7 human adenocarcinoma cells treated with Fe_3_O_4_-PAA–(HP-γ-CDs) nanobioconjugates under SPMHT for 30 min. are presented in Table 4.

As presented in Table 4, the viability of the MCF-7 cell population was significantly affected after exposure to SPMHT for all three concentrations (1, 5 and 10 mg/mL) of the Fe_3_O_4_-PAA−(HP-γ-CDs) nanobioconjugates. The data revealed that the Fe_3_O_4_-PAA−(HP-γ-CDs) sample induced by SPMHT had a viability of only 4.89 ± 0.45% when a concentration of 10 mg/mL was applied.

The results obtained in the current study are more promising compared to those recently obtained by Salimi et al. [38], which revealed that post-magnetic hyperthermia (magnetic field parameters: f = 300 kHz and H = 12 kA/m), the cell viability of MCF-7 breast cancer cells was 36.7% after 24 h. However, Salimi et al. used G4@IONPs nanoparticles (fourth generation of polyamidoamine dendrimer-coated iron oxide nanoparticles) ranging in size between 10 ± 4 nm. Moreover, the concentration of nanoparticles used in the culture medium in this case was 0.5 mg/mL, and the therapy duration was 120 min.

Another result obtained on the MCF-7 breast cancer cells that may be comparable to the ones obtained by us in the current study when referring to the concentration of magnetic nanoparticles of 1 mg/mL, was recently reported by Bhardwaj et al. [12]; the study revealed that nanoparticles of Mn_0.9_Zn_0.1_Fe_2_O_4_ ferrites of size 11.3 nm covered with lauric acid, induced a cell viability of 10% after 24 h when the concentration of nanoparticles was 0.35 mg/mL and the treatment duration was 30 min. for magnetic field parameters of f = 330 kHz and H = 15.3 kA/m.

All the above results show that the viability of MCF-7 cells after magnetic hyperthermia depends on a plethora of factors, such as the type, size and concentration of nanoparticles, the duration of treatment, and the parameters of the applied magnetic field. Therefore, finding all of the optimal conditions for the successful application of magnetic hyperthermia for the complete death of tumor cells with minimal side effects is an essential issue, and is of high interest in the field of alternative cancer therapy.

When applying the external alternating magnetic field, the Fe_3_O_4_ magnetic nanoparticles in the Fe_3_O_4_-PAA–(HP-γ-CDs) nanobioconjugates heat up. The possible mechanisms that leads to the heating of magnetic nanoparticles under magnetic field action are, in this case, Néel–Brown magnetic relaxation [17], the nanobioconjugates being in suspension (culture medium). However, having in view the size of the nanoparticles of ~16 nm and the presence of the organic layer on the surface of nanoparticles [18], the rotation of the nanobioconjugates (Brown relaxation), which also interacts with the cells in the culture medium, is very limited or even blocked [17]. Thus, in this case, the Néel magnetic relaxation mechanism prevails (the rotation of the magnetic moments inside the magnetic nanoparticles), which leads to the heating of the nanoparticles (Figure 6). The required heating temperature in magnetic hyperthermia (42.9 °C) depends on the concentration of nanobioconjugates and the magnetic field applied at the frequency of 312.2 kHz. Once the nanoparticles are heated, the MCF-7 tumor cells located in the culture medium near them or in contact with them, or even containing the nanoparticles, are also heated through the thermal conduction mechanism, as a result of the good thermal conductivity of the medium. Thus, the tumor cells become heated to the temperature of 42.9 °C used in magnetic hyperthermia, which leads to cell death by apoptosis.

Regarding the possible mechanism of action of the nanoparticles within MCF-7 cells, according to Jacob et al. [39], reactive oxygen species (ROS)-based reactions may play a key role in apoptotic processes, which may be further correlated with mitochondrial membrane potential impairment and caspase-3 up-regulation. Furthermore, it is also well-known that the majority of the iron oxide nanoparticles interfere with lysosomal-related signaling pathways [40,41].

According to the results obtained in vitro by us following the application of SPMHT with Fe_3_O_4_-PAA–(HP-γ-CDs) nanobioconjugates on MCF-7 human breast adenocarcinoma cells, the following aspects can be concluded:

(i) The magnetic field parameters implemented in the present study are not cytotoxic on the cell line (MCF-7), as the viability of this line did not decrease below 70%, the limit imposed by ISO standards (70%) [36]; the cellular viability in our case was very high, even at the highest field of 378 Gs (30.08 kA/m in SI units), this being 88.68%;
(ii) The concentrations of magnetic nanoparticles used in the SPMHT experiments and the duration of the therapy are suitable for the effective destruction of MCF-7 tumor cells;
(iii) The Fe_3_O_4_-PAA–(HP-γCDs) sample at a concentration of 10 mg/mL can be considered the sample with the highest in vitro impact, as this concentration induced the most intense cytotoxic effect through SPMHT on MCF-7 human breast adenocarcinoma cells.

The concentrations used by us in the experiment are applicable in magnetic hyperthermia [11,13,15,30,42,43,44,45,46,47]. Furthermore, we previously showed that concentrations up to 10 mg/mL did not produce toxicity on healthy HaCaT human keratinocytes cells [18]; therefore, we used in this in vitro at concentrations of 1, 5 and 10 mg/mL. These concentrations are feasible for the future in vivo application of superparamagnetic hyperthermia, where the concentrations of magnetite nanoparticles in dispersion with very good biocompatibility can even reach 10–20 mg/mL [13,42,44,46,47]. Recently, an even higher concentration value was reported, namely 112 mg/mL, of 12 nm superparamagnetic iron oxide nanoparticles aminosilane-coated in suspension for the therapy of glioblastoma tumors, using a dose of 0.3 mL of magnetic liquid per cubic centimeter of tumor volume [15].

Regarding the bioaccumulation issue of nanoparticles in organs when SPMHT will be tested in vivo, taking into account the approval of the use of the concentration of iron oxide nanoparticles of 12 nm (biocompatible by aminosilane) until approximately 30 mg/mL in clinical trials for MHT [15] (three times higher than the highest dose used by us (10 mg/mL)), we can consider that there will be no major risk of bioaccumulation of nanoparticles in important organs above acceptable toxicity limits.

However, for safety we will consider such evaluations in the future (bioaccumulation of nanoparticles in different organs after nanoparticles injection and superparamagnetic hyperthermia (hepatotoxicity, nephrotoxicity, etc.)) when we conduct in vivo studies.

In summary, by applying SPMHT with Fe_3_O_4_-PAA–(HP-γ-CDs) nanobioconjugates with a concentration of 1, 5 and 10 mg/mL, a high efficacy on the death of MCF-7 breast cancer cells was obtained; the percentages of cancer cells dying were very high in these cases, 88.68, 90.91 and 95.11%, respectively, obtaining an IC_50_ value of 62.88 ± 1 μg/mL.

Considering the efficacy obtained for the destruction of tumor cells, after the application of SPMHT with Fe_3_O_4_-PAA–(HP-γ-CDs) nanobioconjugates, it is important to know what the efficiency is for magnetic hyperthermia with this nanobiomaterial, by determining the intrinsic loss power (ILP) indicator for our nanoparticles, independent of the applied magnetic field. Thus, using the specific loss power (SLP) presented in reference [18], we numerically evaluated its value in the case of our 10 mg/mL sample for the value of a magnetic field amplitude H of 12.73 kA/m and a frequency f of 312.2 kHz, used in the in vitro SPMHT experiment, and found a SLP of 72.2 W/g. By normalizing SLP (SLP/H^2^ f), we determined the value of the ILP indicator of 1.43 nH ⋅m^2^/kg, which indicates that our sample is a very good thermal mediator to efficiently obtain superparamagnetic hyperthermia under the given conditions.

Comparatively, for the bionized nanoferrite (BNF^®^) commercial nanoparticles prepared via the core-shell method with a core of Fe_3_O_4_ and a shell of dextran or hydroxyethyl starch, having a nanoparticle size of 14 nm (close to that of our sample (16 nm)), and for a magnetic field of 9.5 kA/m and a frequency of 614.4 kHz, an ILP of 0.25 nH⋅m^2^/kg was obtained [48]. This value is significantly lower than the one obtained in the case of our sample (1.43 nH⋅m^2^/kg).

However, in the same magnetic field conditions (9.5 kA/m and 614.4 kHz), Darwish et al. [48] reported for ILP a value of 3.8 nH⋅m^2^/kg for core-shell nanoparticles of magnesium iron oxide@tetramethyl ammonium hydroxide (MgIONPs@TMAH), having a size of 15 nm. Moreover, Nishimoto et al. [49] reported an ILP value of ~2 and ~4 for commercial Resovist^®^ and MEADM-033-02 nanoparticles, which are γ-Fe_2_O_3_ nanoparticles coated with carboxydextran and carboxymethyl-diethylaminoethyl dextran, respectively, having a size of 5-6 nm and a concentration of 2 mg-Fe/mL, for a magnetic field of 4 kA/m and 100 kHz.

However, Kandasamy et al. [50] recently showed that in the case of 10 nm nanoparticles of 34DABA-coated SPIOs dispersed in aqueous medium (SPIOs is Fe_3_O_4_, and 34DABA is 3,4-diaminobenzoic acid), there is a dependence of ILP on the concentration of nanoparticles and the applied magnetic field (H and f). The authors showed that the ILP decreased when the concentration increased in the range of 0.5–8 mg/mL, this being attributed to the formation of nanoparticle agglomerates that influenced the magnetic relaxation and decreased the thermal effect; e.g., for 8 mg/mL, the ILP was 0.9 nH m^2^/kg, and for 1 mg/mL, the ILP was 1.7 nH m^2^/kg, under the same magnetic field conditions (10.39 kA/m and 330.3 kHz).

## 4. Conclusions

The use of SPMHT therapy with Fe_3_O_4_-PAA–(HP-γ-CDs) nanobioconjugates, having a mean Fe_3_O_4_ nanoparticle diameter of ~16 nm and a mean nanobioconjugate hydrodynamic diameter of ~20 nm, led to the death of MCF-7 breast cancer cells at a high percentage of 95.11% (cell viability of 4.89%) compared to standard conditions 24 h after treatment. The therapy was carried out for 30 min. at a temperature of 42.9 °C in a harmonic alternating magnetic field with a frequency of 312.2 kHz and an amplitude of 160 Gs (12.73 kA/m), at a concentration of 10 mg/mL of magnetic nanoparticles from nanobioconjugates of Fe_3_O_4_-PAA–(HP-γ-CDs) suspended in the culture medium.

The very good results obtained in our therapy are due to the optimal experimental conditions used for SPMHT and established by us: the optimal size of the Fe_3_O_4_ nanoparticles; the concentration of magnetic nanoparticles; the use of HP-γ-CDs without toxicity to cover the Fe_3_O_4_ magnetic nanoparticles and with a very small thickness; the amplitude and frequency of the magnetic field; and the optimal duration of therapy (without damage to healthy cells).

The percentage of dead cancer cells decreased slightly with a decrease in the concentration of magnetic nanoparticles from nanobioconjugates at 5 mg/mL and 1 mg/mL, namely 90.91 (viability of 9.09%) and 88.68% (viability of 11.32%), respectively, with the percentages remaining at high levels. However, in these two cases, the anticancer therapy remained as effective as with the concentration of 10 mg/mL by repeating the therapy session, or even increasing the duration of the therapy to more than 30 min..

Furthermore, through our in vitro study regarding the safe use of the magnetic field on MCF-7 cells, we showed that the maximum admissible biological limit could be extended to a significantly higher value of ~9.5 × 10^9^ A/m Hz, that is approximately double the value than the one known until now (5 × 10^9^ A/m Hz). This result is very important for the safe practical implementation of SPMHT in vitro and in vivo, which allows the concentration of nanoparticles used to be greatly reduced in magnetic hyperthermia, with a beneficial effect on reducing cytotoxicity.

## Figures and Tables

**Table 1:** Cell viability of the MCF-7 human adenocarcinoma cell line following the application of the magnetic field for a period of 30 min, at a frequency of 312.2 kHz with different amplitudes, after a time interval of 24 h.

Magnetic Field Amplitude (Gs)		160	200	378
Cell viability (%)	Standard conditions (37 °C)	100 ± 0.61	100 ± 0.83	100 ± 0.76
	After applying the magnetic field	99.63 ± 1.20	96.89 ± 1.64	94.50 ± 1.42

**Table 2:** The values of the magnetic field used in therapy depending on the concentration of ferrimagnetic nanoparticles in the samples.

**Concentration of Fe_3_O_4_ nanoparticles (mg/mL)**	1	5	10
**Magnetic field amplitude (Gs)**	378	200	160

**Table 3:** Times necessary to reach the therapy temperature of 42.9 °C, depending on the concentrations of samples.

Sample concentration (mg/mL)	1	5	10
**Duration to reach the therapy temperature 42.9 °C (s)**	1200	150	80

**Table 4:** Data of the cell viability percentages induced by magnetic suspensions under standard conditions (ST) and superparamagnetic hyperthermia (SPMHT) on the human breast adenocarcinoma cell line (MCF-7).

Sample/ Concentration	Cell Viability (%) of MCF-7 Cells under Standard Conditions (ST: 37 °C)	Cell Viability (%) of MCF-7 Cells after SPMHT (42.9 °C, 30 min)
Fe_3_O_4_-PAA–(HP−γ-CDs)/ 1 mg/mL	100 ± 0.80	11.32 ± 0.91
Fe_3_O_4_-PAA–(HP−γ-CDs)/ 5 mg/mL	100 ± 1.02	9.09 ± 0.68
Fe_3_O_4_-PAA–(HP−γ-CDs)/ 10 mg/mL	100 ± 1.40	4.89 ± 0.45

## Data Availability

Not applicable.

## Supplementary Materials

The following supporting information can be downloaded at: https://www.mdpi.com/article/10.3390/pharmaceutics15041145/s1, Figure S1: Schematic representation of IC_50_ parameter using GraphPad Prism software, version 9.3.
