# Supplementary material for: High Efficacy on the Death of Breast Cancer Cells Using SPMHT with Magnetite Cyclodextrins Nanobioconjugates
Source: Pharmaceutics. 2023 Apr 4;15(4):1145. doi: 10.3390/pharmaceutics15041145 (PMC10143435; doi:10.3390/pharmaceutics15041145)
Supplement: Supplementary file 1 [file pharmaceutics-15-01145-s001.zip › pharmaceutics-2249551-supplementary.pdf]

pharmaceutics

Supplementary Material

High Efficacy on the Death of Breast Cancer Cells Using SPMHT with Magnetite Cyclodextrins Nanobioconjugates

Costica Caizer [1], Isabela Simona Caizer-Gaitan [1,2,3], Claudia Geanina Watz [4,5,*], Cristina Adriana Dehelean [5,6], Tiberiu Bratu [2] and Codruța Soica [5,7]

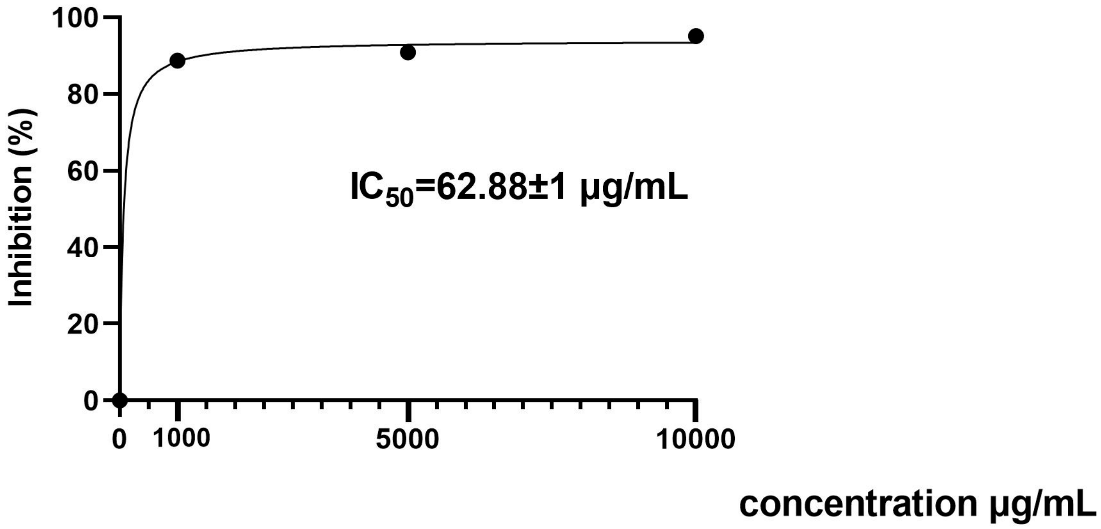

Figure S1. Schematic representation of IC_{50} parameter using GraphPad Prism software, version 9.3.
